# Electrolyte-engineering for enhanced charge storage in Ni metal–organic framework electrodes for high performance supercapacitors

**DOI:** 10.1039/d6ra03346f

**Published:** 2026-06-03

**Authors:** Mrinalini Sharma, Manas Nasit, Sachin, Shruti Lavania, Nitin Kumar Gautam, Nagih M. Shaalan, P. A. Alvi, Ranjeet Kumar Brajpuriya, Aditya Sharma, Shalendra Kumar

**Affiliations:** a Department of Physics, School of Advanced Engineering, UPES Dehradun 248007 India ranjeet.brajpuriya@ddn.upes.ac.in shailuphy@gmail.com; b Department of Physics, College of Science, King Faisal University P.O. Box 400 Al-Ahsa 31982 Saudi Arabia; c Department of Physical Science Banasthali Vidyapith Banasthali Rajasthan 304022 India

## Abstract

The work explores the effects of electrolyte composition and concentration on the electrochemical performance of Ni-MOF electrodes for supercapacitor applications. The synthesized Ni-MOF was structurally and morphologically characterized by XRD, FE-SEM, and XPS. The electrochemical properties were systematically evaluated through CV, GCD, and EIS in alkaline electrolytes of different compositions. Initial investigations were performed in 1 M KOH and 1 M NaOH to assess the effect of electrolyte selection. The Ni-MOF electrode showed a higher electrochemical response in NaOH than in KOH, indicating improved ionic transport and greater utilization of active sites in NaOH. Accordingly, studies were conducted by varying the NaOH concentration (1 M, 3 M, and 5 M). Among these, the 1 M NaOH electrolyte exhibited the max. *C*_s_ obtained from CV analysis of 986.6 F g^−1^, while GCD measurements delivered a *C*_s_ of 433.23 F g^−1^, confirming the enhanced charge storage capability in NaOH electrolyte. To evaluate practical applicability, a Symmetric SC device was constructed using Ni-MOF electrodes in a Swagelok cell with 1 M NaOH electrolyte. The device delivered a *C*_s_ of 31.76 F g^−1^, and 8.75 F g^−1^ from CV and GCD, respectively, with an *E*_d_ of 1.75 Wh kg^−1^ at a *P*_d_ of 85 W kg^−1^ and good cycling stability over 2000 cycles. In addition, a flexible Ni-MOF pouch cell device was fabricated and tested, which exhibited a *C*_s_ of 36.6 F g^−1^ (CV) and 3.24 F g^−1^ (GCD). Ragone plot analysis indicated an *E*_d_ of 0.729 Wh kg^−1^ at a *P*_d_ of 33.75 W kg^−1^. Overall, the results highlight that careful selection and optimization of electrolyte concentration are important for improving the electrochemical behaviour of Ni-MOF-derived supercapacitors.

## Abstract 2

Hydrothermal synthesis of Ni-MOF using trimesic acid and Ni^2+^ enables tunable morphology and structure. The optimized material is integrated into a device, demonstrating enhanced electrochemical performance with molarity variation.

## Graphical abstract

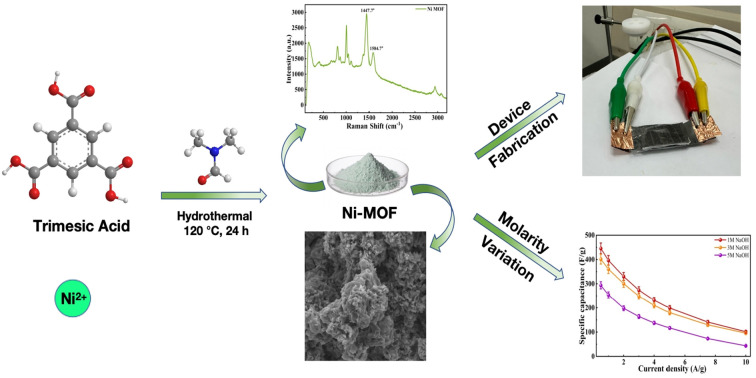

## 1 Introduction

The rapid expansion of industrial activities, with the ongoing growth of the global population, has placed a significant burden on the existing energy resources.^1,2^ Conventional energy systems that depend largely on fossil fuels are inherently limited and are major contributors to global warming and ecological pollution.^3–5^ Thus, the development of sustainable and renewable energy solutions has been recognized as a primary focus of modern scientific research, with the objective of minimizing ecological impact and ensuring long-term energy security.^6,7^ Among the various energy storage systems, supercapacitors (SC) have gained considerable interest due to their high power density, along with quick charge and discharge capabilities, and excellent long-term stability compared with traditional rechargeable batteries.^7–10^ On the basis of that, SC are typically categorized into electric double layer capacitors (EDLCs) and pseudocapacitors, based on their mode of charge storage. In EDLCs, energy storage occurs *via* electrostatic accumulation of ions at the electrode and electrolyte interface, typically using porous carbon-based materials.^11,12^ Although these systems exhibit excellent rate capability and long operational lifetimes, their energy storage capacity is relatively limited.^13–15^ On the other hand, pseudocapacitors store charge through rapid and reversible surface or near-surface faradaic redox reactions, mainly in transition metal oxides or hydroxides.^16,17^ This mechanism allows for a greater capacitance; however, these materials frequently exhibit comparatively lower rate performance and cycling stability.^18,19^ Therefore, the development of high-performance materials used in the electrode that can combine the advantages of both mechanisms has become essential for enhancing the overall performance of the SC.

Metal–Organic Frameworks (MOFs) are considered highly promising materials in recent research for SC electrodes. These materials possess highly tunable structures comprising metal ions coordinated to organic ligands, yielding frameworks with larger surface areas, adjustable pore structures, and redox-active metal centers.^20–22^ Such structural features provide abundant electroactive sites and facilitate efficient ion transport, thereby enhancing both energy and power densities.^23,24^ Moreover, their direct use in electrodes, MOFs can also serve as precursors or templates for producing metal oxides and carbon-based derivatives with improved electrochemical properties.^25,26^ Despite these advantages, the practical applications of many pristine MOFs remain limited due to issues such as structural instability and performance degradation during long-term charge–discharge cycling.^25–29^ Therefore, careful selection of suitable metal centers and organic linkers is necessary to overcome these challenges and improve the durability and electrochemical activity of MOF-based materials.

Extensive studies have been conducted on transition-metal-based MOFs incorporating iron (Fe), cobalt (Co), zinc (Zn), nickel (Ni), and cadmium (Cd) for energy storage applications.^30–36^ Among these, Ni-based MOFs have attracted particular interest due to their favourable redox activity and relatively enhanced theoretical capacitance.^37,38^ As reported by Bashir *et al.*, a pristine Ni-MOF electrode achieved a specific capacitance (*C*_s_) of 411.11 F g^−1^ at a current density (*I*_D_) of 1 A g^−1^, as determined from galvanostatic charge–discharge (GCD) analysis.^39^ Similarly, Wang *et al.* demonstrated that a Ni-MOF employed as a SC electrode delivered a *C*_s_ value of 318 F g^−1^ when tested at 1 A g^−1^, highlighting the moderate electrochemical performance of pristine Ni-based MOF.^40^ Although these studies demonstrate the potential of Ni-MOFs, their *C*_s_ remains moderate due to limited electrical conductivity along with incomplete use of active sites.^41,42^ To further improve the electrochemical behaviour, researchers have focused on modifying the organic linkers to tune the framework structure, enhance electron transport, and increase pore accessibility.^43,44^ For instance, Manyani *et al.* reported that a pristine Ni BDC MOF delivered a *C*_s_ of only 122 F g^−1^ at 0.17 A g^−1^ based on GCD analysis.^45^ Similarly, in another study the same author reported a Ni BTC MOF exhibiting a *C*_s_ of 212 F g^−1^ at 0.42 A g^−1^.^46^ Therefore, the organic linkers play a critical role in regulating structural parameters, electroactive site accessibility and ion diffusion kinetics, which ultimately affect the overall capacitive performance.

The selection and optimization of the electrolyte significantly impact the electrochemical behaviour of SC systems. An appropriate electrolyte selection improves ionic conductivity, enabling faster ion transport and more efficient charge storage.^47–49^ Therefore, the overall conductivity, stability and capacitance of MOF-based electrodes can be improved significantly through the careful selection of both the organic linker and the electrolyte.^43,44,48^ These parameters influence important performance metrics, including *C*_s_, energy density (*E*_d_), power density (*P*_d_), cycling stability and self-discharge behaviour.

The electrolytes used in energy storage devices are generally classified into aqueous, ionic liquid, redox active, organic and solid state systems. Several key factors must be considered, for instance, a wide operating window, low viscosity, low resistivity, good thermal stability, superior ionic conduction, and appropriate concentrations.^50–52^ These characteristics directly affect the series-equivalent resistance (ESR) and the overall power performance of the device. In addition, the efficiency of ion diffusion and long-term cycling stability were influenced by parameters such as electrochemical stability, hydrated ionic radius, and ion–solvent interactions. Although ionic electrolytes and ionic liquids provide a broader operating voltage range and higher *E*_d_, they are often costly and may raise environmental concerns.^53,54^ However, aqueous electrolytes are more economical, environment friendly, and easier to use.^55^ Among them, alkaline electrolytes like KOH and NaOH are widely utilized owing to their high ionic conduction and low viscosity, which promote rapid ion transport and facilitate rapid charge–discharge processes.^56^ Although a smaller hydrated ion radius favors higher ion mobility and enhanced diffusion, electrochemical performance cannot be explained only on the basis of ionic radius. Instead, the overall behavior depends on the fine balance between ionic mobility, electrolyte conductivity and compatibility of the hydrated ion with the electrode structure. Therefore, variations exist in electrochemical performance between NaOH and KOH systems, which are likely governed by the combined effects of the physicochemical parameters.^48,57^ Despite this, a distinct research gap remains in understanding the influence of aqueous electrolyte on Ni-MOF-based materials. Most current studies focus heavily on structural and morphological optimization of the solid-state framework, frequently overlooking the critical role of the chemical nature and molar concentration of the aqueous electrolyte ions within the confined pore spaces, which consequently affect capacitance.

In this work, Ni-MOF was evaluated as a potential electrode material to explore the influence of electrolyte type and concentration on SC performance. The electrochemical characteristics of the Ni-MOF electrode were systematically examined in alkaline electrolytes, where NaOH demonstrated superior capacitive performance compared to KOH. Further optimization of NaOH concentration revealed that 1 M NaOH provided the most favourable electrochemical response. The electrode also exhibited excellent cycling stability, maintaining high capacitive retention during long-term operation. In addition, symmetric SC devices based on Ni-MOF were successfully assembled in both the Swagelok and pouch cell setups, demonstrating stable electrochemical performance along with promising energy and power characteristics, highlighting Ni-MOF for energy storage systems.

## 2 Experimental details

### 2.1 Materials employed

Nickel nitrate hexahydrate (Ni(NO_3_)_2_·6H_2_O, 99% purity, MOLYCHEM), 1,3,5-trimesic acid (BTC, 98% purity, SRL), ethanol, and dimethylformamide (DMF, 99.9% purity, SRL) were all used as precursor materials for the synthesis. Additionally, activated carbon (AC) and polyvinylidene fluoride (PVDF) were employed in electrode preparation. All chemicals were of analytical grade and used as received without any further purification.

All reagents were used as received without any further purification.

### 2.2 Synthesis of Ni-MOF

**Fig. 1:**
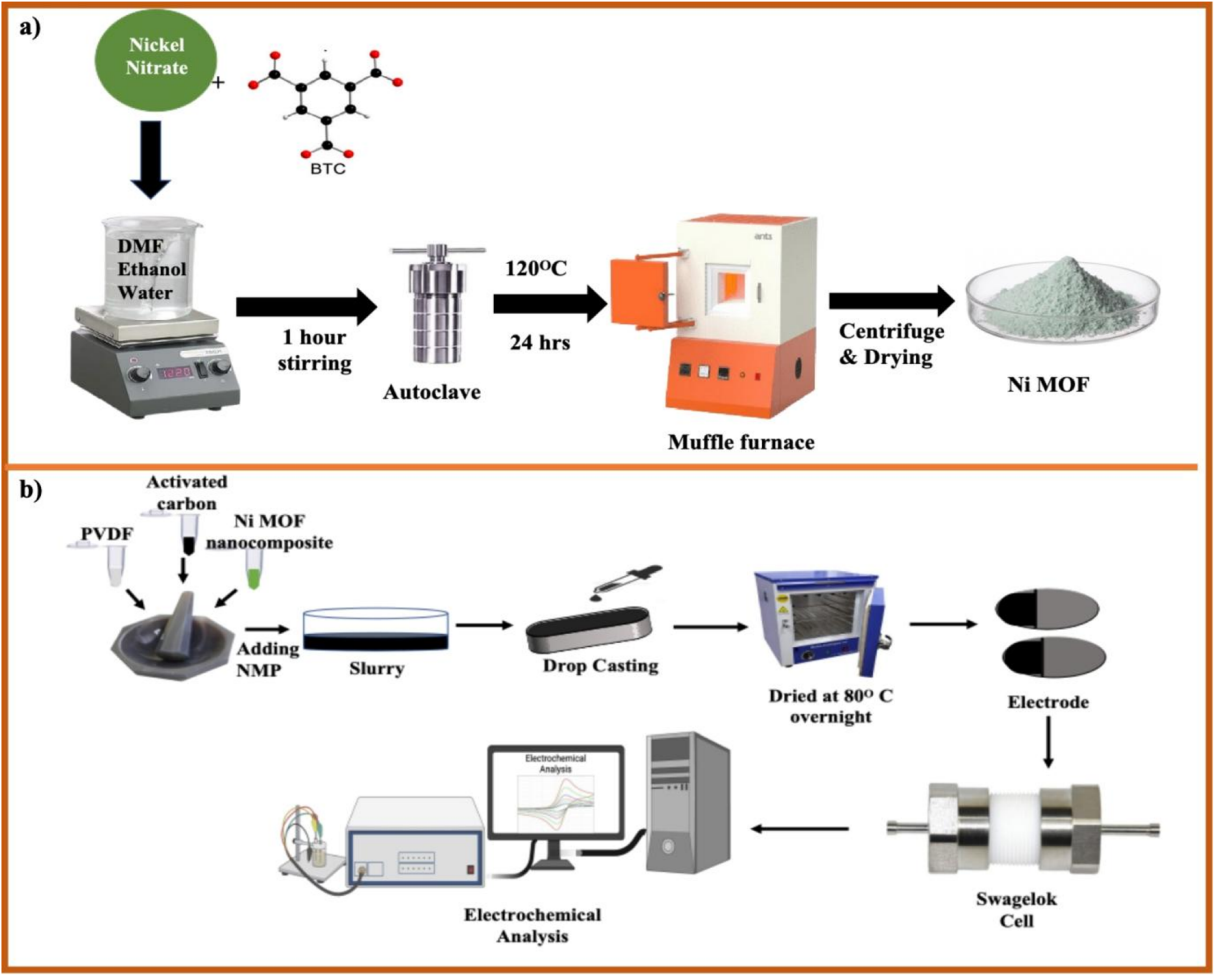
(a) Illustration of the hydrothermal synthesis of Ni-MOF (b) schematic overview of fabrication of Ni-MOF Swagelok cell for electrochemical analysis.

The Ni-based MOF was synthesized *via* a hydrothermal route using BTC as an organic linker. The synthesis procedure of Ni-MOF is schematically illustrated in Fig. 1(a). Initially, 1.163 gm of (Ni(NO_3_)_2_·6H_2_O) was added in solvent volume of 80 mL consisting of 60 mL DMF, 10 mL ethanol and 10 mL DI water, after which 0.14 gm of BTC serving as the organic linker was introduced into the solution. The solution was continuously stirred at 500 rpm for 1 h to obtain a uniform mixture before being transferred to a PTFE-lined stainless steel hydrothermal autoclave. The mixture was heated at 120 °C in a muffle furnace at a heating ramp rate of 5 °C min^−1^ in the autoclave for 24 h under hydrothermal conditions. Thereafter, the obtained precipitate was separated using centrifugation and washed several times with ethanol and DI water to eliminate reactants or remaining solvents. The collected solid product was placed in an oven and dried at 80 °C overnight, resulting in the formation of Ni-MOF.

### 2.3 Fabrication of working electrode

The electrochemical properties of the synthesized Ni-MOF electrode were evaluated in aqueous electrolytes employing a conventional three-electrode setup. Measurements were initially carried out in 1 M KOH and 1 M NaOH, followed by testing in higher concentrations of 3 M and 5 M for both the electrolytes. For the fabrication of working electrode, the active material was thoroughly mixed with AC as conductive agent and PVDF (binder) in a 16 : 2 : 2 weight ratio. The obtained mixture was uniformly ground using a mortar and pestle with gradual addition of few drops of *N*-methyl-2-pyrrolidone (NMP) to achieve a consistent slurry. The resulting paste was uniformly applied over a nickel foam substrate (2 cm × 1.5 cm) and then dried in a hot air oven at 80 °C for 12 h, as shown in Fig. 1(a).

### 2.4 Fabrication of electrodes for Swagelok-type symmetric supercapacitor device

The electrode was prepared using the drop-casting method in which the Ni-MOF was combined with PVDF as a binder and AC as a conductive additive in a mass ratio of 80 : 10 : 10 to improve the mechanical integrity and electrical conductivity. A few drops of NMP were introduced into the mixture, which was then stirred thoroughly to obtain a homogeneous paste. The resulting paste was carefully deposited over a circular graphitic paper serving as current collector for electrode fabrication, which was then kept at 80 °C for 12 h in a hot air oven to completely remove the residual solvent. The symmetric SC was then prepared using a Swagelok type cell, with Whatman filter paper serving as the separator. Before assembling the SC, the separator was soaked in an aqueous electrolyte consisting of 1 M NaOH. The device was referred to as the Ni-MOF symmetric SC, as illustrated in Fig. 1(b).

### 2.5 Fabrication of electrodes for symmetric supercapacitor (pouch cell configuration)

The pouch cell electrode was fabricated through a process similar to that discussed earlier. More specifically, the slurry mixture was deposited on a rectangular piece of graphite paper as the electrode material, while polyvinyl NaOH (PVA-NaOH) was used as the separator and electrolyte. To prepare the gel electrolyte, 5.5 gm each of PVA and NaOH were dissolved in 45 mL DI water under constant stirring at 70 °C for approximately 10 h, resulting in a clear homogeneous solution. The resulting solution was cast into a Petri dish to achieve the required separator thickness and dried overnight at room temperature, forming a solid gel electrolyte.

For pouch cell assembly, the two electrodes were placed on either side of the prepared gel electrolyte. The assembled cell, referred to as the Ni-MOF pouch cell, and its electrochemical performance were evaluated using a two-electrode setup *via* CV, GCD, and EIS.

### 2.6 Characterization of the Ni-MOF

The crystal structure of the sample was analyzed through powder X-ray diffraction (PXRD) on a Bruker D8 Advance EcoPro diffractometer with Cu Kα radiation operating at a wavelength of 1.5406 Å. Fourier transform infrared spectroscopy (FTIR) was performed on a PerkinElmer spectrometer to identify the functional groups and chemical bonds present in the materials. In addition, X-ray photoelectron spectroscopy (XPS) was employed to examine the surface chemistry and elemental oxidation states using a Kratos Analytical AXIS SUPRA+ system with a monochromatic Al Kα X-ray source at 225 Watts. The morphology and structural features were analyzed using field emission scanning electron (FESEM) using a JEOL JSM-7900F model. The textural analysis of the material was done using Brunauer–Emmett–Teller (BET) analysis involving nitrogen adsorption desorption process performed at 77 K temperature using an Anton Paar Autosorb 6100 FKM MP-AG instrument which provided the detailed information about the surface area along with pore size characteristics of the sample. Further, a Corrtest (CS2350M) electrochemical workstation was used to investigate the electrochemical characteristics of the synthesized electrodes.

### 2.7 Electrochemical analysis

A conventional three-electrode setup was used to evaluate the electrochemical behaviour of the Ni-MOF electrode. The measurements were performed in aqueous electrolytes of 1 M KOH and 1 M NaOH, using a Ni-MOF coated on a nickel substrates employed as the working electrode, while the reference and counter electrodes were Ag/AgCl and platinum wire, respectively. A Corrtest (CS2350M) electrochemical workstation was used for all studies, including Cyclic Voltammetry (CV), GCD and Electrochemical Impedance Spectroscopy (EIS) to investigate the electrochemical characteristics of the electrode. CV measurements were recorded over a potential window of 0–0.9 V at scan rate (SR) up to 100 mV s^−1^, while the GCD tests were conducted at *I*_d_ up to 10 A g^−1^ to examine the charge–discharge characteristics under varying conditions. The EIS measurements were carried out using frequencies varying between 100 kHz and 10 mHz. The electrochemical parameters, including *C*_s_, *E*_d_ and *P*_d_ were calculated using standard electrochemical equations.1
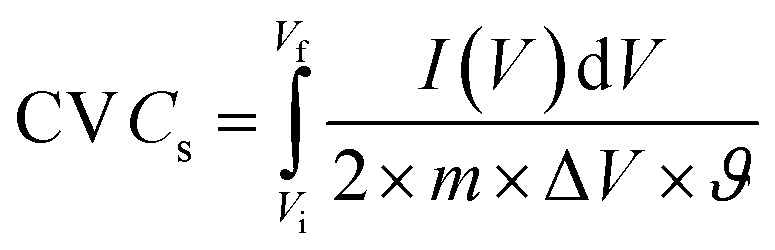
2
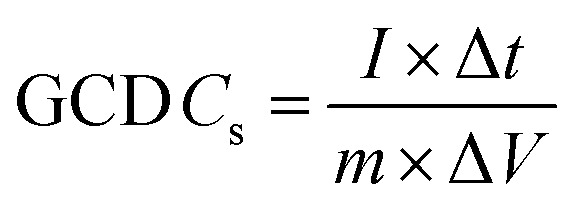
3
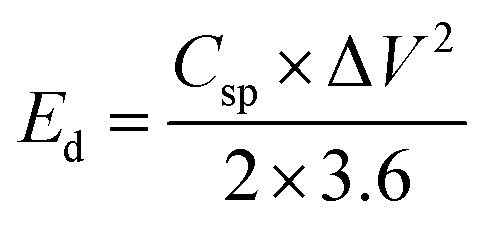
4
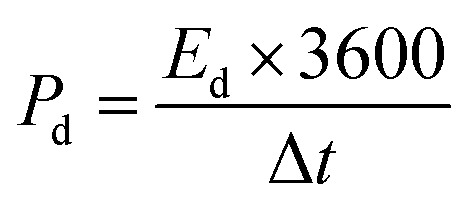
Here, *C*_s_ (F g^−1^) represents the specific capacitance, Δ*V* corresponds to the operating potential window, and 
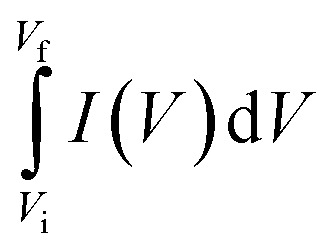
 denotes the total area enclosed by the CV curve. The terms *I* and *m* refers to the applied current and total active material mass, respectively, while *ϑ* denotes the SR and Δ*t* corresponds to the discharge time. Using these relations, the performance parameters of the symmetric SC device including *C*_s_ (F g^−1^), *E*_d_ (Wh kg^−1^) and *P*_d_ (W kg^−1^) were determined.5
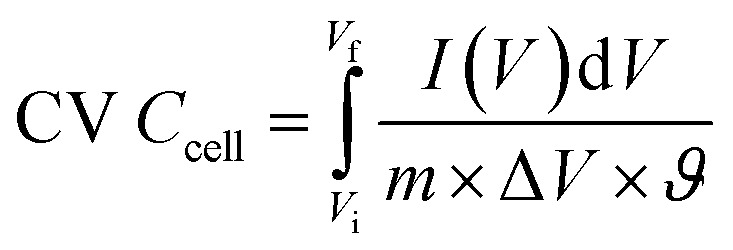
6
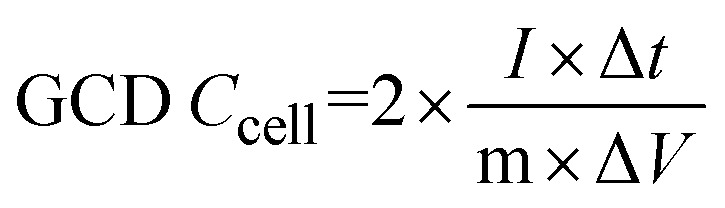
7
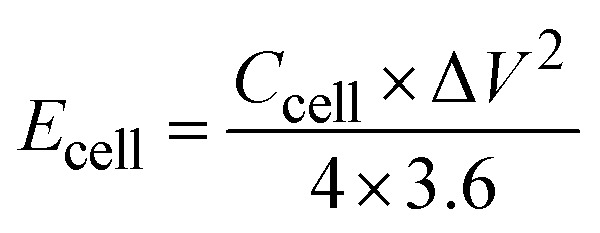
8
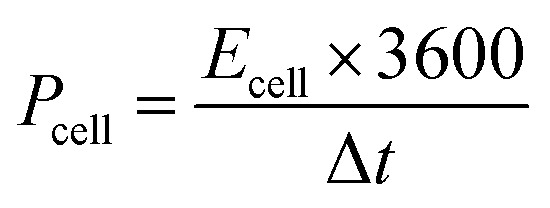


## 3 Results and discussion

### 3.1 Characterization of powder sample

**Fig. 2:**
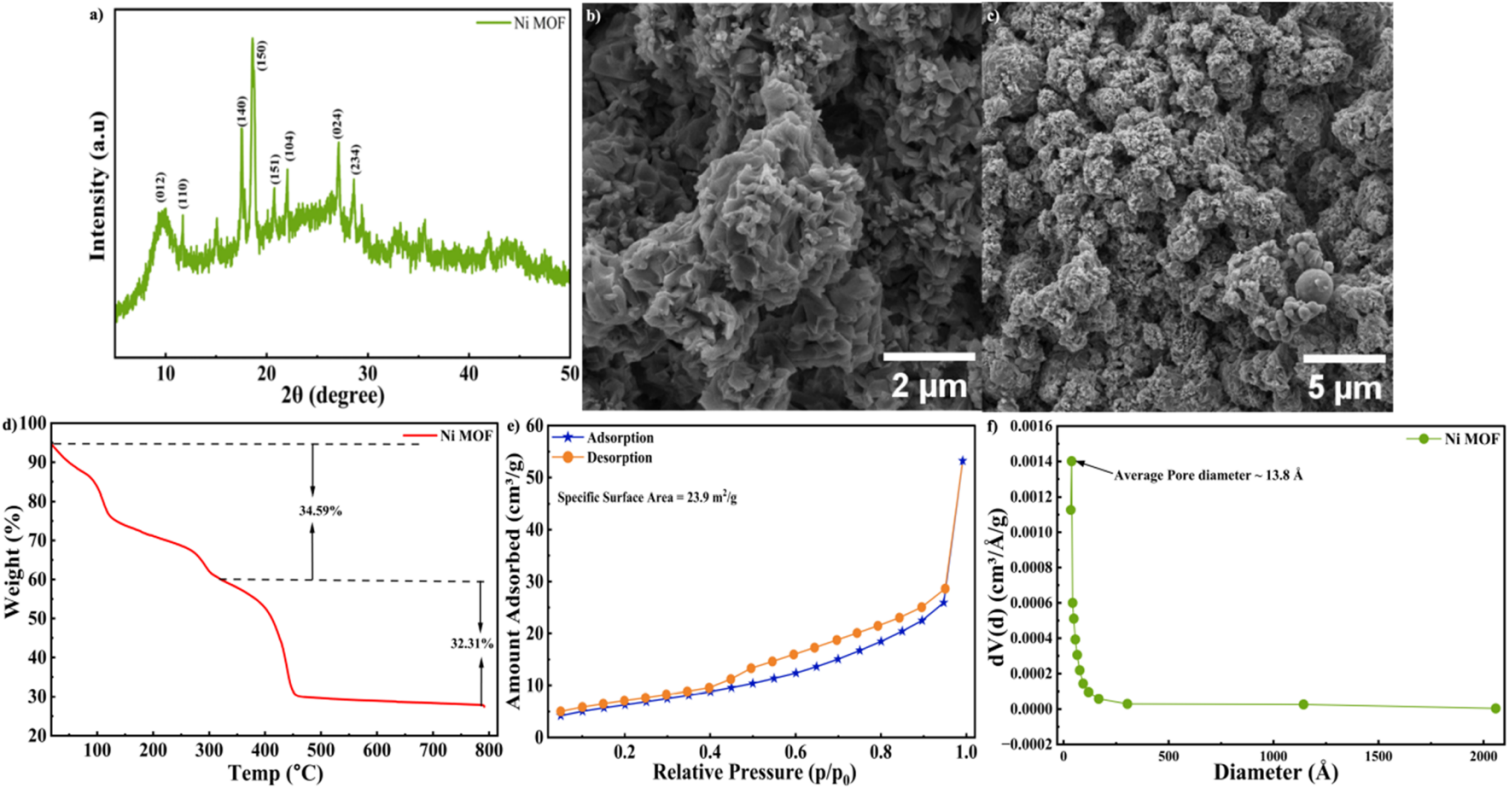
(a) PXRD sample of Ni-MOF (b), (c) SEM images at 2 µm and 5 µm magnifications of Ni-MOF, respectively (d) TGA profile (e) BET isotherm analysis (f) pore size distribution.

The phase purity and crystallinity of the synthesized Ni-MOF were investigated by PXRD, as shown in Fig. 2(a). The diffraction peaks appearing at 11.69°, 17.51°, 18.7°, 20.7°, 22.05°, 27.11°, and 28.6° correspond to the (110), (140), (150), (151), (104), (024), and (234) planes, respectively. The observed PXRD pattern shows good agreement with previously reported Ni-MOF patterns in the literature, indicating successful formation of the crystalline framework.^58^ A comparison with the simulated PXRD pattern (CCDC 738061) is shown in Fig. 2(a), which shows good agreement in peak position.^59^ The absence of additional impurity peaks further confirms the phase purity of the synthesized material. These results indicated that the Ni-MOF was successfully prepared without any impurity. The morphology of the Ni-MOF was analyzed through FE-SEM and the corresponding micro graphs were shown in Fig. 2(b) and (c). The images revealed that the material was composed of densely arranged micro structures across the surface. These structures were constituted by the aggregation of multiple thin nanosheets that assemble together to form three-dimensional clusters. The overall architecture resembled the morphology of a Tagetes (marigold) flower like morphology, where petal like sheets were arranged radially, resulting in a flower shaped appearance. At higher magnification, the clusters appear to be composed of layered and interlinked sheet-like structures. The nanosheets overlap and stack with each other, generating a highly textured surface. This hierarchical arrangements results in the creation of interconnected pores and voids within the structure, forming a porous network throughout the material. This morphology offered expanded surface region with multiple active centers, thereby improving the interaction at the electrode–electrolyte junction. To examine the functional groups within the Ni-MOF, the material was analysed *via* FT-IR and shown in Fig. S1(a). The absorption band around 3418 cm^−1^ was due to the O–H stretching vibrations, which suggests the potential existence of hydroxide functional groups within the Ni-MOF framework. The peaks at 1622 cm^−1^ and 1439 cm^−1^ correspond to the asymmetric and symmetric vibration of carboxylate functional groups, respectively. Additionally, the peak at 1383 cm^−1^ corresponds to the symmetric vibrations of the COO^−^ function. The presence of this band, along with similar IR absorption features in the range of 1350–1650 cm^−1^, confirms the coordination of carboxylate moieties with Ni ions within the MOF framework.^58^ The peaks observed in the region of 720–650 cm^−1^ were related to C–H bending vibrations within the MOF framework.^59^ In Raman Spectroscopy, vibrational modes that are associated with the metal centers were typically observed in the low frequency region, particularly between 100 and 600 cm^−1^. The bands observed between 150 and 500 cm^−1^ corresponds to Ni–O vibrational modes as presented in Fig. S1(b). A notable band around 500 cm^−1^ indicated the presence of stretching of the Ni–O coordination bond, confirming the interaction between the nickel ions and the organic ligand. The bands around 813 and 865 cm^−1^ belong to out-of-plane ring bending vibration, with the latter attributed to the out of plane bending of aromatic C–H groups present in the synthesized MOF structure. Peaks at 1006 and 1045 cm^−1^ are indicative of C=C stretching vibrations in the benzene ring of the organic linker. Furthermore, the spectral features in the range of 1446.5–1584.7 cm^−1^ arise from the symmetric and asymmetric stretching vibrations of the carboxylate groups. These vibrations indicate the successful coordination of the carboxylate moieties with the metal centers, supporting the formation of a stable metal–ligand framework.^59^ To study the optical behavior of the prepared Ni-MOF, UV-vis diffuse reflectance spectroscopy (UV-DRS) was performed. As shown in Fig. S1(c), the absorption spectrum showed two absorption peaks at 396.2 nm and 659.5 nm. The former is caused by metal charge transfer (LMCT) transition, while the latter is due to the d–d transitions of Ni(ii), which was consistent with previously reported studies. In addition, a weak absorption band observed near 259 nm was associated with the π–π* electronic transition of the organic ligand. The optical band gap of the material was estimated using the Tauc relation:

9 (*αhν*)^*n*^ = *β*(*hν* − *E*_g_)

**Fig. 3:**
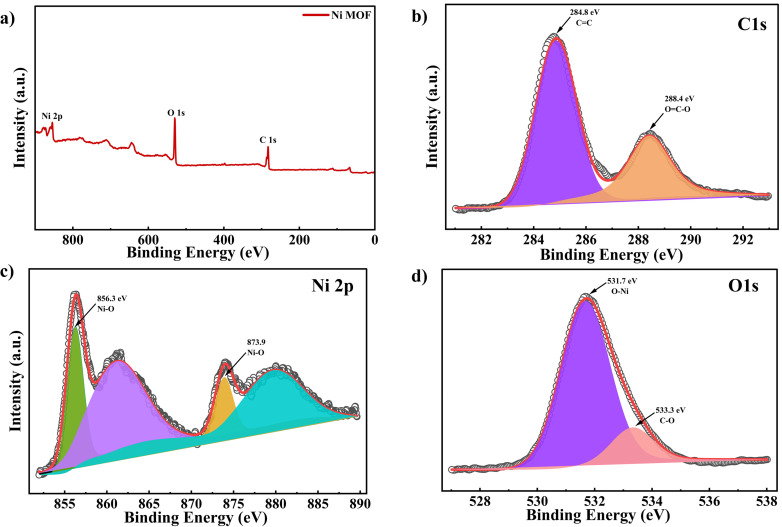
XPS analysis of Ni-MOF (a) survey spectra (Su) (b) high resolution C 1s spectra (c) Ni 2p spectra and (d) O 1s spectra.

In this case *α* represents the coefficient of absorption, whereas *β* and *hν* stands for a constant and the photon energy, respectively. The value of band gap (*E*_g_) was obtained from the graph between (*αhν*)^*n*^ and *hν*, where the parameter *n* varies with the type of electronic transition, taking values of 2 for direct transitions 0.5 for indirect transitions. From the Tauc plot shown in Fig. S1(d), the direct *E*_g_ of the Ni-MOF was determined to be approx. 3.82 eV, suggesting that the material may also possess potential for optical applications.^46^ Thermogravimetric analysis (TGA) was conducted to evaluate the composition and stability of the Ni-MOF, as presented in Fig. 2(d), by monitoring the variation in sample mass with rising temperature. This method is widely used for MOF's to understand their thermal response, such as the release of guest molecules, degradation of organic linkers, and the eventual formation of metal oxide residues at elevated temperatures. The TGA profile of the Ni-MOF sample exhibits three primary stages of mass loss. The initial phase occurs below 122 °C, resulting in an approximate weight reduction of 17.2%. This initial loss was primarily due to the removal of moisture and remaining solvent content retained within the framework's pores. The second stage reached approximately 318.3 °C and involved an additional mass loss of about 15.92%. This gradual decrease in weight loss was due to the loss of water molecules tightly bound to the framework, potentially *via* hydrogen bonding interactions. A significant reduction in weight occurred within the range of 320 °C to 450 °C, corresponding to thermal decomposition of the BTC linker.^60^ The deterioration of the ligand disrupts the coordination network, ultimately resulting in the structural breakdown of the Ni-MOF framework at temperatures above 450 °C. Previously reported Ni based MOFs synthesized by L. Jin *et al.* showed thermal stability only up to 400 °C.^61^ W. W. Lestari *et al.* reported a major weight loss in the temperature range of 260–405 °C due to decomposition of the organic linker, after which the framework completely decomposed into NiO above 400 °C.^62^ Similarly, another report by K. Karuppiah *et al.* stated that ligand breakdown resulted in the collapse of the Ni-BTC framework around 400 °C.^63^ In comparison, the present Ni-MOF exhibited better thermal stability and structural integrity at elevated temperatures. The porosity and surface characteristics of the synthesized Ni-MOF were assessed through Nitrogen (N_2_) adsorption–desorption measurements as shown in Fig. 2(e), which was utilized to determine its specific surface area. Fig. 2(f) depicts the pore size distribution of the synthesized Ni-MOF. The isotherm curve exhibits a characteristic type 4 profile with a reversible hysteresis loop, signifying the existence of a mesoporous structure.^46^ The Ni-MOF exhibited a specific surface area of 23.9 m^2^ g^−1^, as determined by BET method. The relatively large surface area enhanced interaction at the electrolyte and the electrode interface, providing additional active sites for electrochemical reactions. This improved interface promotes swift ion diffusion and effective electron transport, thereby enhancing the electrochemical behaviour of the electrode material. The pore size distribution indicated that the average pore diameter was 13.8 nm, thereby confirming the mesoporous characteristics of the material. The presence of mesopores along with a substantial surface area is essential in electrode materials, as these structural characteristics facilitate the efficient transport of ions and electrons during electrochemical reactions. XPS was carried out to determine the oxidation states and constitute elements in the synthesized Ni-MOF. As depicted in Fig. 3(a), the survey spectrum (Su) confirms that the sample is mainly composed of Ni, O, and C elements. The high resolution C 1s spectrum in Fig. 3(b) revealed two characteristic peaks at binding energies (BE) of 284.8 eV and 288.4 eV, which can be assigned to C=C and C–O functional groups, respectively. The Ni 2p spectrum exhibited two prominent peaks at BE of 856.3 eV and 873.9 eV, corresponding to the Ni 2p_3/2_ and Ni 2p_1/2_ as presented in Fig. 3(c). The spin–orbit energy difference between these peaks were about 17.6 eV, which was characteristic peak of nickel species. Additionally, satellite peaks appeared at approx. 861.3 eV and 879.8 eV. The presence and separation of these peaks suggests that nickel mainly exists in +2 oxidation state in the Ni-MOF framework. The slightly higher BE values compared to earlier reports may arise from strong coordination between Ni^2+^ ions and the COO^−^ groups of the organic linker. Furthermore, the O 1s spectrum shown in Fig. 3(d) exhibited two clear peaks, indicating that oxygen atoms were present in different chemical environments. The peak centered around 533.3 eV was linked with oxygen in the carboxylate (C–O) group, while the 531.7 eV peak corresponded to oxygen involved in metal–ligand coordination between nickel and the organic linker.^59^

### 3.2 Electrochemical behaviour of Ni-MOF employing a conventional three-electrode configuration

**Fig. 4:**
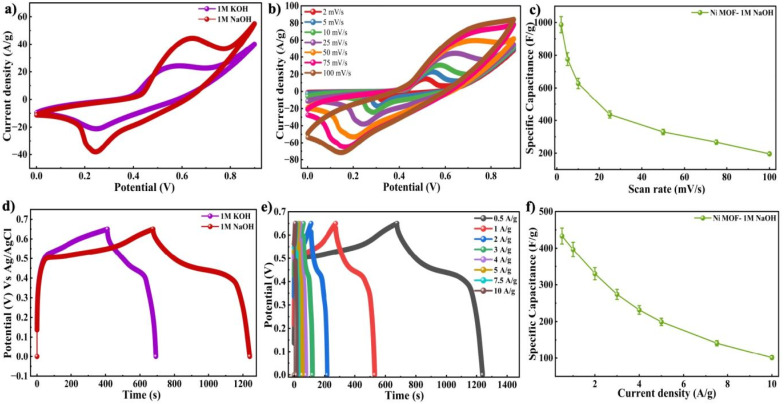
(a) Comparison CV curves of 1 M KOH and 1 M NaOH at 25 mV s^−1^ (b) CV curves in 1 M NaOH (c) *C*_s_*vs.* SR graph in 1 M NaOH (d) comparison GCD graph of 1 M KOH and 1 M NaOH at 0.5 A g^−1^ (e) GCD graph in 1 M NaOH (f) *C*_s_*vs. I*_D_ in 1 M NaOH.

The electrochemical properties of the Ni-MOF were systematically investigated in two alkaline electrolytes, 1 M KOH and 1 M NaOH, to understand the influence of electrolyte ions on its charge storage characteristics. The CV behaviour of the synthesized Ni-MOF electrodes in both the electrolytes, with the measurements conducted over a range of 0–0.9 V at SR from 2 to 100 mV s^−1^. The Ni-MOF electrode exhibits distinct redox peaks, indicating the presence of reversible faradaic reactions and verifying the pseudocapacitive characteristics of the material.^64^ Notably, the CV profiles retain their characteristic shape even at higher SRs with only slight distortion. This behaviour suggests that the electrode structure was capable of rapid charge movement and improved ion mobility within the electrode structure. To further evaluate the effect of electrolyte cations, the electrochemical behaviour of the Ni-MOF electrode in KOH and NaOH electrolytes were compared in Fig. 4(a) at 25 mV s^−1^. Fig. 4(b) shows the CV curves of Ni-MOF in 1 M NaOH.

Under identical conditions, the Ni-MOF electrode exhibited a significantly higher *C*_s_ in 1 M NaOH reaching 986.6 F g^−1^ at a SR of 2 mV s^−1^ as presented in Fig. 4(c), compared to 680 F g^−1^ in 1 M KOH. The area under the CV curve recorded in NaOH was significantly larger than that in KOH, suggesting that the NaOH electrolyte has a superior charge storage capacity. These observations clearly suggest that NaOH provides more favourable electrochemical conditions for the Ni-MOF electrode.

The variation in performance was due to the hydration characteristics and cation size in the electrolyte. The interaction with the electrode surface and their transport behaviour within the porous structure were influenced by the fact that the K^+^ (0.31 nm) has a slightly smaller hydrated radius compared to Na^+^ (0.4 nm). The ion mobility and diffusion kinetics can be influenced by variations in the hydrated ionic radius, which in turn affects the charge storage process. Furthermore, the intrinsic ionic radii of the cations are also significant in the process of charge transport. The K^+^ ion (0.138 nm) has a slightly larger ionic radius than Na^+^ (0.102 nm), which may restrict its effective diffusion and accessibility to active sites within the electrode material. In addition, factors such as ion–electrode interaction, electrolyte wettability, pore accessibility, and redox kinetics significantly determine the overall electrochemical behaviour. Therefore, the Ni-MOF exhibited better electrochemical performance in NaOH compared to KOH due to improved ion diffusion and redox interactions within the porous structure.^65^

The electrode's capacitive properties were assessed through GCD measurements. The GCD results were consistent with the nonlinear characteristics of the curves, which are typical for pseudocapacitive materials. The measurements were performed within the potential window of 0.6 to 0.65 with *I*_D_ ranging from 0.5 A g^−1^ to 10 A g^−1^. Fig. 4(d) shows the comparison of discharge times at 0.5 A g^−1^ was illustrated, where the electrode tested in 1 M NaOH demonstrates a longer discharge duration, which suggests a higher *C*_s_. The GCD graph of Ni-MOF in 1 M NaOH was presented in Fig. 4(e). The *C*_s_ values calculated from the GCD curves were plotted as a function of *I*_D_ in Fig. 4(f). In comparison to the KOH electrolyte, the Ni-MOF electrode demonstrated a significantly superior electrochemical behaviour, achieving a maximum *C*_s_ of 433.23 F g^−1^ over a potential range of 0.65 V at 0.5 A g^−1^.

**Fig. 5:**
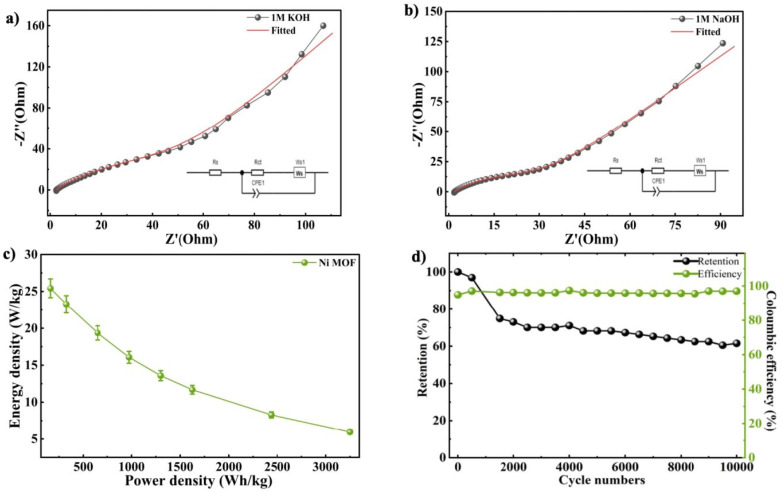
EIS graph of the Ni-MOF electrode in (a) 1 M KOH and (b) 1 M NaOH (c) Ragone plot and (d) cyclic stability measured in 1 M NaOH.

To further conduct the analysis of the charge transport behaviour and interfacial properties within the Ni-MOF electrode, EIS measurements were performed. It is an effective technique for evaluating resistance components and ion diffusion processes that influence the performance of the supercapacitor. The Nyquist plots of the Ni-MOF electrodes were obtained in the KOH and NaOH electrolytes using the frequency range of 100 kHz to 0.01 Hz. The plots exhibited two distinct regions, a semicircular arc at high frequencies and an inclined straight line at low frequencies, where the semicircle reflects the charge transfer resistance at the electrode and electrolyte junction during oxidation–reduction processes, while the linear portion reflects ion diffusion and capacitive behaviour within the electrode material.^66^ The diameter of semicircle is related to charge transfer resistance (*R*_ct_), while interception at high frequency refers to the solution resistance (*R*_s_). The *R*_s_ and *R*_ct_ for the Ni-MOF electrode were found to be 2.35 Ω and 86.49 Ω, respectively, in 1 M KOH as presented in Fig. 5(a). In comparison, the electrode exhibited lower resistance values in 1 M NaOH, with *R*_s_ and *R*_ct_ recorded as 1.922 Ω and 38.22 Ω as shown in Fig. 5(b), indicating improved charge transfer and better electrochemical performance in the NaOH electrolyte. Furthermore, the *E*_d_ and *P*_d_ were employed using the standard equations as discussed earlier, where the Ni-MOF electrode achieved 162.5 W kg^−1^ of *P*_d_ and 25.42 Wh kg^−1^ of *E*_d_ when operated in 1 M NaOH, as displayed in Fig. 5(c). Additionally, the Ni-MOF electrode was evaluated for long-term stability to investigate its cycling performance. The electrode tested in 1 M NaOH exhibited excellent durability, maintaining coulombic efficiency of nearly 97.04%, followed by 10 000 cycles of charging and discharging, as presented in Fig. 5(d). This outcome suggests that the material exhibited strong structural stability and electrochemical behaviour during prolonged operation.

These results clearly highlight the superior energy storage capability of the electrode in NaOH electrolyte. A summary of the *C*_s_ values at different current densities along with the corresponding *E*_d_ and *P*_d_ of NaOH electrolyte is provided in Table 1.

**Table 1:** *C*_s_ variation with SR for the prepared Ni-MOF electrode in 1 M KOH and 1 M NaOH

Electrolyte	Variation of *C*_s_ (F g^−1^) values with SR (mV s^−1^)						
	2	5	10	25	50	75	100
1 M KOH	680	500.62	389.4	267.7	190.11	148.5	122.3
1 M NaOH	986.6	776.5	627.9	435.9	329.4	266.9	195.9

### 3.3 Electrochemical analysis of Ni-MOF at different electrolyte molarities using a three-electrode setup

**Fig. 6:**
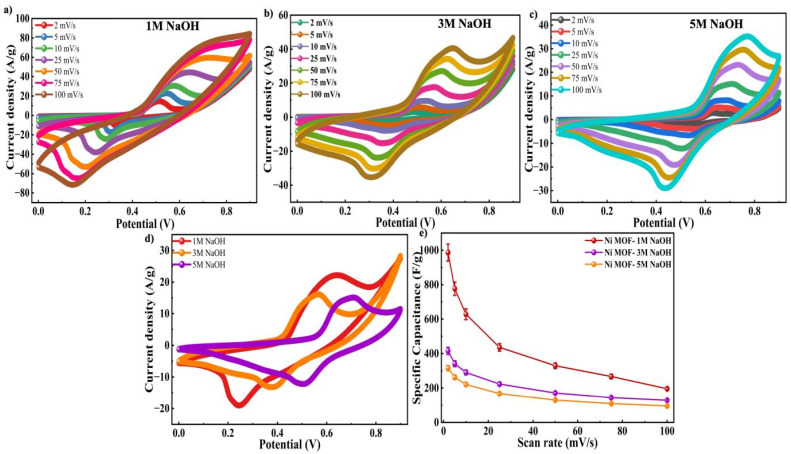
CV profiles of the Ni-MOF electrode in (a) 1 M NaOH (b) 3 M NaOH (c) 5 M NaOH (d) comparison graph of 1 M NaOH, 3 M NaOH and 5 M NaOH at 25 mV s^−1^ of Ni-MOF (e) *C*_s_*vs.* SR graph of 1 M NaOH, 3 M NaOH and 5 M NaOH of Ni-MOF.

As the Ni-MOF demonstrated enhanced electrochemical activity in NaOH electrolyte, further studies were performed by varying the electrolyte concentration to 1 M, 3 M and 5 M NaOH at various SRs, as shown in Fig. 6(a)–(c). To evaluate the influence of electrolyte molarity on the electrochemical response, CV measurements were conducted at a SR of 25 mV s^−1^, and the corresponding curves were presented in Fig. 6(d). Among the tested electrolytes, Ni-MOF electrode operating in 1 M NaOH demonstrated significantly higher current response compared to those recorded in 3 M and 5 M NaOH. This improvement was recorded due to increased enclosed area of the CV curve, indicating a higher *C*_s_. The calculated *C*_s_ values for the Ni-MOF electrode were 986.6 F g^−1^, 415.5 F g^−1^, 315.2 F g^−1^ at 2 mV s^−1^ over a voltage range of 0.9 V, respectively, as presented in Fig. 6(e). For gaining a better insight into the characteristics of charge storage, Dunn's method was applied to analyze the CV data. This method enables the differentiation of current contributions that result from surface capacitive processes and diffusion controlled reactions that take place within the bulk of the material. The correlation between current and SR can be expressed as:

10 *I* = *I*_c_ + *I*_d_ = *av*^*b*^

11 log *I* = *b* log *v* + log *a*

In this analysis, *I* represents the sum of all current responses, *I*_c_ and *I*_d_ refer to the current response of the capacitive-controlled process and the diffusion-controlled process, respectively.^67^ The relationship between *I* and SR was examined through log(*I*) *versus* log(*v*) plot, where the slope of the fitted line provided the *b* value. This parameter helped in identifying the dominant charge storage mechanism. A *b* value close to 1 indicated a capacitive-controlled process, whereas a value near 0.5 suggests a diffusion-controlled mechanism.^68^ These values lying between these limits indicated the coexistence of both processes. The corresponding log(*I*)–log(*v*) plots for the Ni-MOF electrodes in KOH, NaOH, and their different molar concentrations were presented in Fig. S2(a). The calculated *b* values were found to be 0.41 for 1 M KOH and 0.60 for 1 M NaOH. For higher NaOH concentrations of 3 M and 5 M, the *b* values were 0.68 and 0.66, respectively. The results suggests that the charge storage mechanism was a result of synergistic effect of surface-controlled capacitive behaviour and diffusion-driven faradaic reactions. To further understand the charge storage process at the interface of electrode–electrolyte system, the peak current obtained from CV measurements carried out under different SR's. This approach allowed for a cleared estimation of the relative contributions of each processes contributed more significantly, while at higher SR's. It was observed that at lower SR's, diffusion-controlled processes contributed more significantly, while at higher SR's, surface-controlled reactions became dominant. As presented in Fig. S2(b–e) this trend indicated that ion transport became increasingly surface-limited at higher SR's, confirming that both mechanisms worked together to define the overall electrochemical behaviour of the Ni-MOF electrode.

**Fig. 7:**
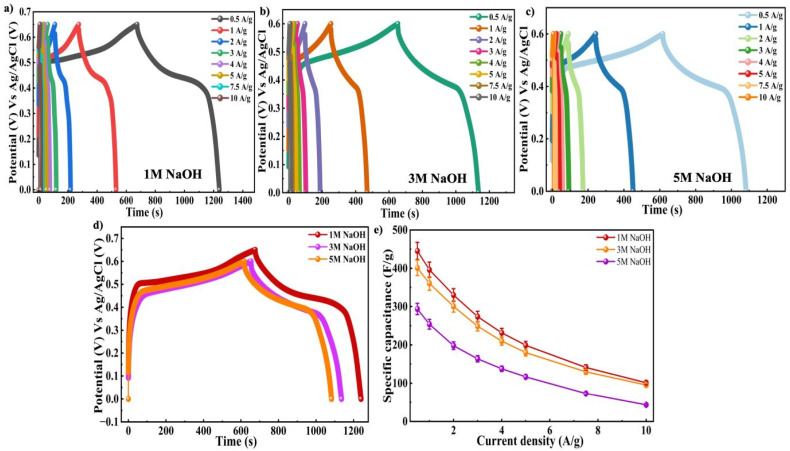
GCD curves of (a) 1 M NaOH (b) 3 M NaOH (c) 5 M NaOH (d) comparison graph of 1 M NaOH, 3 M NaOH and 5 M NaOH at 25 mV s^−1^ of Ni-MOF (e) *C*_s_*vs. I*_D_ of 1 M NaOH, 3 M NaOH and 5 M NaOH of Ni-MOF.

To examine the charge storage properties, GCD were performed at a *I*_D_ of 0.5 A g^−1^ for various NaOH concentrations, and their corresponding profiles were shown in Fig. 7(a)–(c). It was observed that the potential window differed slightly with electrolyte concentration. In the case of 1 M NaOH, the stable potential window was recorded to be 0.65 V, whereas for 3 M and 5 M NaOH the potential window was limited to 0.6 V. This reduction at higher molarities could be due to increased ionic strength and enhanced side reactions at elevated electrolyte concentrations, which facilitates faster and more reversible redox reactions which results in earlier onset of electrolyte decomposition or polarization effects. As a result, in order to maintain electrochemical stability during measurements, a slightly narrower potential range was selected.^69,70^ The electrode tested in 1 M NaOH exhibited the longest charge–discharge duration, implying improved charge storage capability compared to higher concentration of electrolytes as shown in Fig. 6(d). The observed increase in effectiveness may be explained by better ion mobility and utilization of active sites for redox processes, as well as optimal ion transfer in the selected concentration of electrolyte. The calculated *C*_s_ values were 433.23 F g^−1^ within a potential range of 0.65 V and 401.3 F g^−1^ and 387.7 F g^−1^ for 1 M, 3 M and 5 M NaOH within 0.6 V range, respectively as presented in Fig. 6(e). These results clearly demonstrate that 1 M NaOH provides the most suitable electrochemical environment for the Ni-MOF electrodes, leading to enhanced charge storage.

**Fig. 8:**
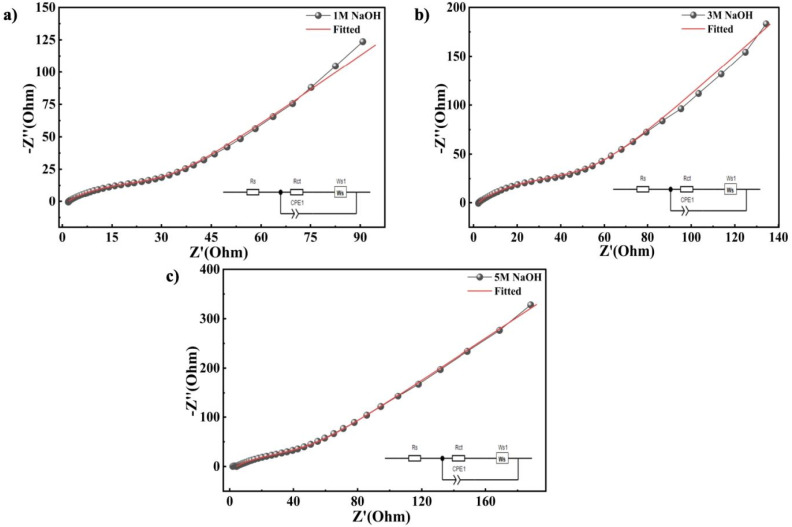
Fitted EIS graph of Ni-MOF (a) 1 M NaOH (b) 3 M NaOH (c) 5 M NaOH.

EIS was employed to investigate the charge storage behaviour and charge transfer processes at the Ni-MOF electrode interface. The Nyquist plots recorded at different NaOH concentrations of 1 M, 3 M and 5 M, which showed clear differences in impedance response. Notably, the electrode tested in 1 M NaOH displays the smallest semi-circle at high frequencies, indicating lower resistance and more efficient charge transfer at the junction of electrode and the electrolyte. The impedance parameters revealed that the Ni-MOF electrode in 1 M NaOH exhibited lower *R*_s_ and *R*_ct_ values. In contrast, higher charge transfer resistance values were observed for 3 M (*R*_s_ = 2.30 Ω, *R*_ct_ = 70.74 Ω) and 5 M NaOH (*R*_s_ = 4.05 Ω, *R*_ct_ = 86.28 Ω) as displayed in Fig. 8(a)–(c). The increase in resistance (*Z*′) with increasing NaOH was mainly attributed to the porous nature of the Ni-MOF electrode and electrolyte properties. The Ni-MOF electrode showed the lowest *R*_s_ and *R*_ct_ values in 1 M NaOH, indicating an efficient ion diffusion and quick charge trasfer at the electrode–electrolyte interface. At higher electrolyte concentrations (3 M and 5 M NaOH), the ionic strength and viscosity of the solution were increased, which decreased the mobility of ions and slowed the diffusion of OH^−^ ions into the porous framework of the electrode. The electrochemical performance of Ni-MOF was highly dependent on electrolyte penetration into the porous channels and accessibility to active redox sites, and thus the limited ion transport at higher NaOH concentrations resulted in slower electrochemical kinetics and higher *R*_ct_. In addition, higher concentrations lead to stronger ion–ion interactions and possible pore blockage which further contributed to the increase in resistance. Hence, the larger semicircle were observed in the Nyquist plots for 3 M and 5 M NaOH. Overall the results indicate that 1 M NaOH offers the most favourable conditions for enhanced electrochemical activity and faster kinetics in Ni-MOF.

A detailed comparison of the electrochemical parameters obtained from CV, GCD and EIS measurements in NaOH electrolytes with concentrations of 1 M, 3 M and 5 M were summarized in Table 2. Furthermore, Table 3 illustrates the detailed electrochemical properties of the Ni-MOF with existing literature, highlighting the significance of the current results. On comparing with the earlier reported Ni based MOFs, the synthesized Ni-MOF showed better capacitance performance along with significantly improved long term cycling stability. For instance, Ni MOF-H_2_BDC electrodes previously reported a *C*_s_ of 432 F g^−1^, but the capacitance retention decreased to 60.48% after 5000 cycles.^71^ Similarly, several Ni MOF-BTC systems exhibited lower *C*_s_ values ranging from 212 to 283 F g^−1^ with comparatively limited cycling performance. Ni-BTB and Ni-MOF-BDC microflower structures also demonstrated lower electrochemical performance despite the use of other alkaline electrolytes.^66,72^ The improved electrochemical behaviour of the present Ni-MOF can be due to its large surface area, porous framework, enhanced accessibility of electroactive sites, efficient ion transport pathways, and favourable redox activity of Ni centers within the structure. In addition, the hydrothermal synthesis approach may have contributed to better structural stability during repeated charge–discharge processes, resulting in improved capacitance retention over prolonged cycling.

**Table 2:** Influence of NaOH concentration (1 M, 3 M and 5 M) on the CV, GCD and EIS behaviour of Ni-MOF electrodes

Electrolyte molarity (NaOH)	CV (at 2 mV s^−1^)	GCD (at 0.5 A g^−1^)	Resistance	
			*R*_s_	*R*_ct_
1 M	986.6 F g^−1^	433.2 F g^−1^	1.92 Ω	38.22 Ω
3 M	415.5 F g^−1^	401.3 F g^−1^	2.30 Ω	70.74 Ω
5 M	315.2 F g^−1^	387.7 F g^−1^	4.05 Ω	86.28 Ω

**Table 3:** Electrochemical performance comparison of Ni-MOF electrodes synthesized *via* different methods and evaluated in various electrolyte molarities

S. no	Material	Electrolyte molarity	Synthesis	Specific capacitance (F g^−1^)	Cyclic stability	Ref.
1	Ni MOF-H_2_BDC	2 M KOH	Hydrothermal	432	60.48% (5000 cycles)	71
2	Ni MOF-BTC	1 M LiOH	Hydrothermal	230	∼85% (1000 cycles)	46
3	RGO-Ni MOF-BTC	6 M KOH	—	64.3	80% (2000 cycles)	73
4	Ni MOF-BTC nanospheres	1 M KOH	Hydrothermal	283	154% after 1000 cycles	46
5	Ni MOF-BTC nanospheres	1 M NaOH	Hydrothermal	212	∼85% (1000 cycles)	46
6	Ni-MOF-BDC microflowers	1 M KOH	Solvothermal	196.24	—	66
7	Ni-MOF-BDC microflowers	1 M Na_2_SO_4_	Solvothermal	158.11	—	66
8	Ni-BTB	6 M KOH	Hydrothermal	156	Near 100% (10 000 cycles)	72
9	Ni-MOF	1 M NaOH	Hydrothermal	433.23	97.04% (10 000 cycles)	This work

### 3.4 Electrochemical analysis of a symmetric Ni-MOF Swagelok type supercapacitor device

**Fig. 9:**
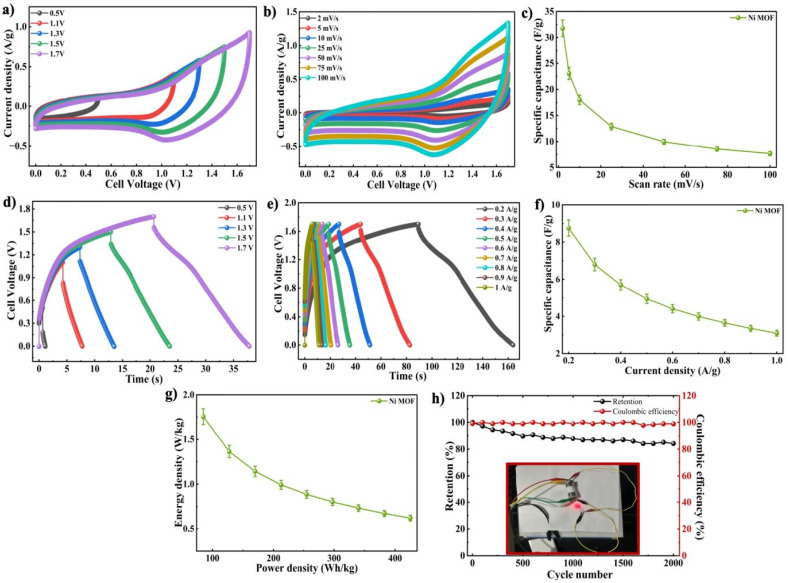
(a) Operating voltage optimization using CV (b) CV profiles of Ni-MOF Swagelok device (c) *C*_s_*vs.* SR graph (d) operating voltage optimization using GCD (e) GCD plot Ni-MOF Swagelok device (f) variation of *C*_s_ with *I*_D_ (g) Ragone analysis of Ni-MOF Swagelok device; inset: LED illumination by Ni-MOF Swagelok device. (h) Cyclic stability of Ni-MOF Swagelok device.

Moreover, the potential application of the Ni-MOF electrode was investigated through the preparation of a symmetrical SC based on a Swagelok cell design after the determination of the electrochemical performances of the material in 1 M NaOH solution in a three-electrode system. The device was composed of two Ni-MOF electrodes which were identical and separated by a porous separator. The electrochemical characteristics of the fabricated cell was further examined *via* 1 M NaOH electrolyte using CV and GCD measurements. The operating voltage range was determined through CV measurements conducted over a potential range of 0.5–1.7 V at a SR of 100 mV s^−1^, as illustrated in Fig. 9(a). The broader potential window observed for the Swagelok cell, in comparison to the Ni-MOF electrode in varying molarities (1 M, 3 M and 5 M) can be primarily due to the variation in electrochemical testing configuration. Furthermore, the electrochemical behaviour of the Ni-MOF was evaluated *via* a three-electrode setup, where the potential window was limited to 0.9 V to avoid electrolyte decomposition and to maintain stable and reversible redox reactions. However, the Swagelok device was tested in a two-electrode configuration, where the total cell voltage was distributed between both electrodes. This allowed the assembled device to be stable over a wider voltage range of 1.7 V. In addition, the combined contribution of both the electrodes and the stable electrolyte environment helped the device achieve an extended operating voltage window without significant polarization effects. The curves were observed to have an approximately quasi-rectangular shape, with a slight distortion at higher potentials. As the SRs increased, the area of CV gradually expanded along with a rise in current response, indicating improved charge storage behaviour. Without the presence of clearly defined redox peaks, the slight deviation from the rectangular shape implies that the surface controlled Faradaic reactions, as well as EDLC, contributed to the hybrid charge storage mechanism. The CV responses of the symmetric device were observed at various SRs between 2 to 100 mV s^−1^ and their *C*_s_ values were calculated and summarized as displayed in Fig. 9(b). The symmetric cell delivered a maximum *C*_s_ of 31.76 F g^−1^ at a SR of 2 mV s^−1^. With increasing SR, the capacitance gradually decreased to 23 F g^−1^ at 5 mV s^−1^, and further to 18 F g^−1^, 12.8 F g^−1^, 9.94 F g^−1^, 8.54 F g^−1^ and 7.65 F g^−1^ at SRs of 10 mV s^−1^, 25 mV s^−1^, 50 mV s^−1^, 75 mV s^−1^ and 100 mV s^−1^, correspondingly as displayed in Fig. 9(c). The measured decrease in capacitance at elevated SRs can be due to kinetic limitations in ion transport, where ions were not able to fully access the internal structure of the Ni-MOF within a short time period. As a result, the charge storage becomes restricted to the active sites from the surface. However, the CV profiles maintained across all SRs which indicated enhanced rate performance and stable electrochemical behaviour of the Ni-MOF electrodes when employed in a symmetric Swagelok cell.

Further, the suitable operating potential window was investigated *via* GCD measurements of the Ni-MOF Swagelok cell over a potential of 0.5–1.7 V, as displayed in Fig. 9(d). Fig. 9(e) shows the GCD curves that were recorded within a similar voltage range used for the CV measurements, at *I*_D_'s ranging from 0.2 to 1 A g^−1^. The charge–discharge curves displayed a nearly symmetric triangular shape, suggesting efficient energy retention along with a reversible electrochemical process within the device. However, the small charge–discharge time associated with the Ni-MOF Swagelok device compared to that of the Ni-MOF electrodes in varying NaOH molarities was primarily ascribed to the distinction between electrochemical setups. The NaOH molarity studies were performed using a three-electrode system, the intrinsic electrochemical behaviour of the active material was evaluated with this type of system and was less polarized with generally longer discharge times. While the Swagelok device was tested in a two-electrode configuration and all of anode and cathode correspondingly contributed to the cell performance leading relatively higher internal resistance and short discharge times. Therefore, the reduced time scale observed in the GCD curves of the assembled device reflects its practical device-level electrochemical behaviour.

The *C*_s_ values were calculated using eqn (7), and the results were summarized in Fig. 9(f). The symmetric device delivered a max. *C*_s_ of 8.75 F g^−1^ at *I*_D_ of 0.2 A g^−1^. As the applied *I*_d_ increased, the capacitance gradually decreased. The capacitance values recorded at 0.3 A g^−1^, 0.4 A g^−1^, 0.5 A g^−1^, 0.6 A g^−1^, 0.7 A g^−1^, 0.8 A g^−1^, 0.9 A g^−1^ and 1 A g^−1^ showed a steady decline with increasing *I*_D_. This reduction in capacitance at higher *I*_D_'s can be attributed to the rise in internal resistance and sufficient time for electrolyte ions to assess the inner electroactive regions of the electrode.^74^ As a result, the material's outer surface primarily contributes effectively to charge storage at higher *I*_D_'s which limits the overall capacitive performance. The significant higher *C*_s_ values observed in the three-electrode configuration compared to the symmetric device was mainly due to the difference between ideal half-cell testing and practical full-cell operation. In the three-electrode system, the electrochemical properties of the Ni-MOF electrode were examined individually under controlled conditions, which allowed better utilization of the electroactive sites. In contrast, the symmetric two-electrode device experienced additional limitations such as internal resistance, electrode polarization, restricted operating voltage, and slower ion transport, all of which reduced the overall capacitance. Moreover, since Ni-MOF is a pseudocapacitive material, the faradaic redox reactions are not fully utilized in the symmetric configuration, leading to comparatively lower *C*_s_ values in the full-cell device. The energy storage capability of the device was analyzed with respect to *E*_d_ and *P*_d_ by Ragone plot method, as demonstrated in Fig. 9(g). The Swagelok type cell demonstrated suitability for efficient energy storage applications, which achieved a max. *P*_d_ of 85 W kg^−1^ and an *E*_d_ of 1.75 Wh kg^−1^. Furthermore, the Ni-MOF electrode's long durability was examined through cycling stability measurements. The device was subjected to 2000 successive charging and discharging cycles at *I*_D_ of 1 A g^−1^, maintaining a coulombic efficiency of approximately 99% and retention about 84.25%, as displayed in Fig. 9(h). A marginal reduction in capacitance value upon prolonged cycling resulted from several factors, including partial irreversibility of the redox reaction, deterioration of the electrolyte, loss of the active materials, as well as possible heat effects arising from cycling. These processes can gradually limit the transportation of ions and decrease the count of electrochemically available active sites. However, the device maintained consistent cycling behaviour throughout the test, despite its minor decline. Fig. S3(a–f) showed the FE-SEM images of the electrode and after 2000 charge–discharge cycles at different magnifications. The FE-SEM analysis revealed that the electrode possessed a porous morphology composed of uniformly distributed Ni-MOF along with conductive additives and binder materials such as activated carbon and PVDF across the electrode surface. The porous structure facilitated efficient electrolyte penetration and enhanced charge transfer kinetics, which contributed to improved electrochemical performance. A comparison of the FE-SEM images before and after cycling indicated only minor surface modifications after 2000 cycles. No significant surface roughening, particle fracture, or structural collapse was observed, suggesting that the electrode maintained good structural integrity and stability during prolonged electrochemical cycling.^75^

The Swagelok device was assembled in order to operate a red LED bulb that functioned for about 1.5 min, as shown in the Fig. 9(h). This result validates the suitability of the Ni-MOF electrode material for energy storage applications.

### 3.5 Electrochemical analysis of a symmetric Ni-MOF pouch cell supercapacitor device

**Fig. 10:**
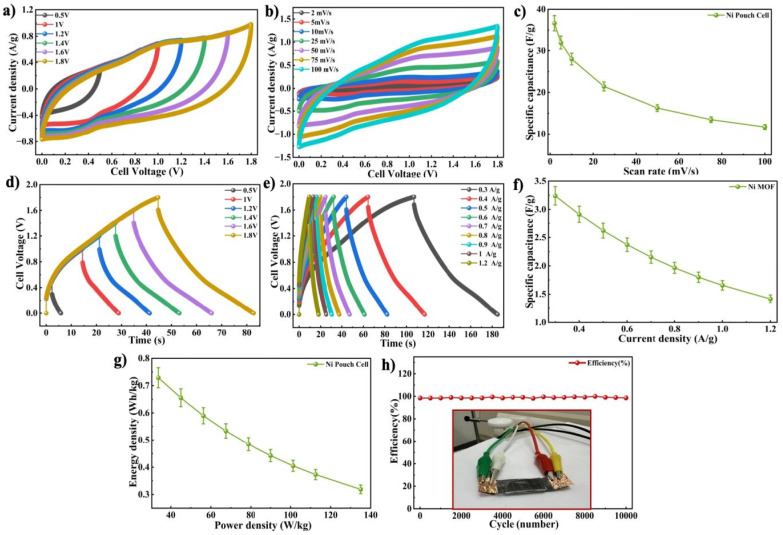
(a) Operating voltage optimization using CV (b) CV curve of Ni-MOF pouch cell device (c) *C*_s_*vs.* SR graph (d) operating voltage optimization using GCD (e) GCD plot Ni-MOF pouch cell device (f) variation of *C*_s_ with *I*_D_ (g) Ragone Plot of Ni-MOF pouch cell, inset: Ni-MOF pouch cell (h) cyclic stability of Ni-MOF pouch cell.

**Fig. 11:**
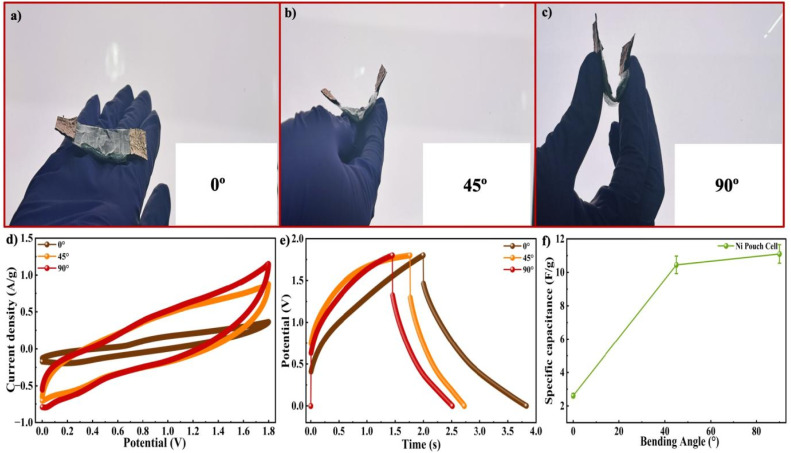
Bending tests of Ni-MOF Pouch cell at (a) 0° (b) 45° (c) 90° (d) comparison graph of CV curves at various bending angles (e) comparison graph of GCD curves at various bending angles (f) *C*_s_*vs.* Bending angle graph of Ni-MOF pouch cell.

The electrochemical properties of the Ni-MOF pouch cell were further examined in a two electrode setup using CV, GCD and cycling stability measurements. To determine the suitable operating voltage range of the device, CV measurements were conducted at a SR of 50 mV s^−1^, while gradually increasing the applied potential from 0.5 to 1.8 V, as presented in Fig. 10(a). Accordingly, potential windows of 0.5, 1, 1.2, 1.4 V, 1.6 V and 1.8 V were chosen for subsequent electrochemical studies. The CV responses of the pouch cell within specific voltage ranges were measured at SRs ranging from 2 to 100 mV s^−1^, as shown in Fig. 10(b). The Ni-MOF pouch cell delivered a *C*_s_ of 36.6 F g^−1^ at 2 mV s^−1^ (SR), as presented in Fig. 10(c). At higher SR, a steady rise in *I*_D_ response was observed, indicating increase charge transfer and capacitive characteristics. The behaviour showing charge–discharge of the pouch cell was further analyzed using GCD measurements performed at *I*_D_'s between 0.3 and 1.2 A g^−1^ within the optimal potential range, as shown in Fig. 10(d and e). The resulting GCD curves obtained as a result showed typical characteristics of a capacitive nature along with reversible discharge and charging behavior. The *C*_s_ values obtained based on these curves were presented in Fig. 10(f). The device achieved its peak *C*_s_ of 3.24 F g^−1^ at 0.3 A g^−1^, while maintaining better capacitive performance at higher *I*_D_'s, reflecting high rate capability. Furthermore, the energy storage capability of the pouch cell was assessed through *E*_d_ and *P*_d_ by Ragone plot analysis, as illustrated in Fig. 10(g). The *E*_d_ and *P*_d_ of the Ni-MOF pouch cell were determined to be 0.72 Wh kg^−1^ and 33.75 W kg^−1^, respectively, proving its applicability as a flexible energy storage device. The extended stability of the pouch cell was assessed by subjecting the device to 10 000 successive charging and discharging cycles, while monitoring the coloumbic efficiency. As presented in Fig. 10(h), the device achieved high cycling durability, maintaining approximately 98% of initial capacitance after extended cycling. This result indicated that the pouch cell maintained a stable electrochemical performance during repeated operation.^76^ To further demonstrate its practicability, the fabricated Ni-MOF pouch cell was successfully employed to illuminate a red LED which lasted for 25–40 seconds. The structural bendability of the symmetric pouch cell was also examined by measuring CV responses when bent at 0°, 45° and 90°, as presented in Fig. 11(a–c). The CV and GCD profiles calculated at a SR of 100 mV s^−1^ and *I*_D_ at 1.2 A g^−1^, maintained their overall shape with minimal distortion even when subjected to mechanical deformation, suggesting that it maintained good electrochemical stability during bending as displayed in Fig. 11(d) and (e). The measurements were carried out at a high *I*_D_ to evaluate the rapid charge–discharge capability and mechanical stability of the device under practical conditions. Due to the high *I*_D_, the charging and discharging processes occurred within a shorter time span, which resulted in the observed time scale in the GCD curves. Interestingly, the *C*_s_ increased from 2.61 F g^−1^ at 0° to 10.54 F g^−1^ at 45°, and further to 11.1 F g^−1^ at 90°, as demonstrated in Fig. 11(f) This improvement may be due to enhanced electrolyte contact and better utilization of active sites when the device was bent. Overall, these results confirmed that the Ni-MOF based pouch cell achieved good mechanical robustness along with stable electrochemical performance, highlighting its potential for use in flexible and wearable energy storage systems.

## 4 Conclusion

The present work highlights the fabrication of the Ni-MOF through hydrothermal method and its further deposition on nickel foam to investigate its electrochemical performance for SC applications. The crystal structure and phase composition of the synthesized material were invesygated through XRD, with the characteristic peak observed at 18.70°, which confirmed the formation of a highly ordered crystalline Ni-MOF structure. The diffraction pattern exhibited distinct peaks associated with crystallographic planes of Ni-MOF, indicating good crystalinity and phase purity. The surface morphology was studied using SEM, which revealed a flower-like morphology resembling Tagetes petals formed by the agglomeration of micro-sized particles. This morphology offers a large surface area along with an adequate number of accessible active sites, thereby enhancing the electrochemical performance and efficient transportation of ions. In addition, XPS analysis confirmed the presence of Ni^2+^ species along with carbon and oxygen elements associated with the organic ligand framework. The electrochemical behaviour of the Ni-MOF electrode were systematically studied by conducting CV, GCD, and EIS analyses under alkaline conditions by using KOH and NaOH. In terms of electrochemical performance, NaOH showed better results than KOH. Further investigations were carried out using different NaOH concentrations (1 M, 3 M and 5 M), where 1 M NaOH exhibited the most favourable electrochemical performance. The Ni-MOF electrode delivered a high *C*_s_ of 986.6 F g^−1^ from CV measurements and 433.23 F g^−1^ from GCD analysis in 1 M NaOH, while higher electrolyte concentrations resulted in comparatively lower capacitance values. The electrode also achieved excellent cycling stability, maintaining approximately 98% coulombic efficiency after prolonged charging and discharging cycles. To explore practical applicability, a symmetric type Swagelok SC device was fabricated using Ni-MOF electrodes and 1 M NaOH. The device achieved *C*_s_ values of 31.7 F g^−1^ (CV) and 8.75 F g^−1^ (GCD), with the max. *E*_d_ of 1.75 Wh kg^−1^ at a *P*_d_ of 85 W kg^−1^. Furthermore, a flexible Ni-MOF pouch cell device was also constructed, which demonstrated stable electrochemical performance and retained about 84.25% of its capacitance after 2000 cycles. Overall, the results underscore that the choice of electrolyte and its concentration plays a significant role in determining the electrochemical behaviour of Ni-MOF electrodes, demonstrating their potential for advanced energy storage systems.

## Conflicts of interest

The authors declare that they do not have any known competing financial interests or personal relationships that could have appeared to influence the work reported in this paper.

## Data Availability

The data used to support the findings of this study are included in the article. Raw data that support the findings of this study are available from the corresponding author upon reasonable request.

## References

[cit1] Jie H., Khan I., Alharthi M., Zafar M. W., Saeed A. (2023). Util. Policy.

[cit2] Omer A. M. (2008). Renew. Sustain. Energy Rev..

[cit3] Arutyunov V. S., V Lisichkin G. (2017). Russ. Chem. Rev..

[cit4] Kalair A., Abas N., Saleem M. S., Kalair A. R., Khan N. (2021). Energy Storage.

[cit5] Höök M., Tang X. (2013). Energy Policy.

[cit6] Valentine S. V. (2011). Renew. Sustain. Energy Rev..

[cit7] Gayen D., Chatterjee R., Roy S. (2024). Int. J. Environ. Sci. Technol..

[cit8] Olabi A. G., Abbas Q., Al Makky A., Abdelkareem M. A. (2022). Energy.

[cit9] Wang L., Wen L., Tong Y., Wang S., Hou X., An X., Dou S. X., Liang J. (2021). Carbon Energy.

[cit10] Lavania S., Aljaafari A., Dhiman G., Gautam N. K., Nasit M., Dalela S., Alvi P. A., Brajpuriya R. K., Sharma A., Kumar S. (2026). J. Phys. Chem. Solids.

[cit11] Shao H., Wu Y.-C., Lin Z., Taberna P.-L., Simon P. (2020). Chem. Soc. Rev..

[cit12] Zhang L. L., Gu Y., Zhao X. S. (2013). J. Mater. Chem. A.

[cit13] Naoi K., Ishimoto S., Miyamoto J., Naoi W. (2012). Energy Environ. Sci..

[cit14] Miller E. E., Hua Y., Tezel F. H. (2018). J. Energy Storage.

[cit15] Sun J., Luo B., Li H. (2022). Adv. Energy Sustain. Res..

[cit16] Nguyen T., Montemor M. de F. (2019). Advanced Science.

[cit17] Khot M., Kiani A. (2022). Int. J. Energy Res..

[cit18] Augustyn V., Simon P., Dunn B. (2014). Energy Environ. Sci..

[cit19] Anwar A. W., Majeed A., Iqbal N., Ullah W., Shuaib A., Ilyas U., Bibi F., Rafique H. M. (2015). J. Mater. Sci. Technol..

[cit20] Naithani S., Kumar P., Dubey R., Thetiot F., Layek S., Goswami T., Kumar S. (2025). J. Mater. Chem. C.

[cit21] Xu G., Zhu C., Gao G. (2022). Small.

[cit22] Wei Y.-S., Zhang M., Zou R., Xu Q. (2020). Chem. Rev..

[cit23] Jia X., Liu C., Neale Z. G., Yang J., Cao G. (2020). Chem. Rev..

[cit24] Kong L., Zhong M., Shuang W., Xu Y., Bu X.-H. (2020). Chem. Soc. Rev..

[cit25] Li Y., Xu Y., Yang W., Shen W., Xue H., Pang H. (2018). Small.

[cit26] Hwang J., Ejsmont A., Freund R., Goscianska J., Schmidt B. V. K. J., Wuttke S. (2020). Chem. Soc. Rev..

[cit27] Gao H., Shen H., Wu H., Jing H., Sun Y., Liu B., Chen Z., Song J., Lu L., Wu Z., Hao Q. (2021). Energy Fuels.

[cit28] Gao X., Dong Y., Li S., Zhou J., Wang L., Wang B. (2020). Electrochem. Energy Rev..

[cit29] Kunwar N., Naithani S., Goswami T., Dubey R., Layek S., Kumar S., Mangalam J. (2026). Coord. Chem. Rev..

[cit30] Umezawa S., Douura T., Yoshikawa K., Tanaka D., Stolojan V., Silva S. R. P., Yoneda M., Gotoh K., Hayashi Y. (2023). Energy Environ. Mater..

[cit31] Zheng S., Li X., Yan B., Hu Q., Xu Y., Xiao X., Xue H., Pang H. (2017). Adv. Energy Mater..

[cit32] Ramasubramanian B., Chinglenthoiba C., Huiqing X., Xiping N., Hui H. K., Valiyaveettil S., Ramakrishna S., Chellappan V. (2022). Surf. Interfaces.

[cit33] He Y., Yin Z., Wang Z., Wang H., Xiong W., Song B., Qin H., Xu P., Zeng G. (2022). J. Mater. Chem. A.

[cit34] Rong H., Liu Z., Gao G., Su L., Chen X., Huang H., Liu W., Liu Q. (2025). J. Alloys Compd..

[cit35] Khokhar S., Anand H., Chand P. (2022). J. Energy Storage.

[cit36] Hangarter C. M., Dyatkin B., Laskoski M., Palenik M. C., Miller J. B., Tyagi M., Klug C. A. (2024). J. Energy Storage.

[cit37] Helal A., Shaheen Shah S., Usman M., Khan M. Y., Aziz Md. A., Mizanur Rahman M. (2022). Chem. Rec..

[cit38] Dinesh B., Saravanan N., Kumar A. S. (2022). Chem. Eng. J. Adv..

[cit39] Bashir M. I., Imran M., Anjum F., Nasir A., Taimur S., Baig F., Zaheer Z., Qasim F. (2025). Solid State Commun..

[cit40] Wang H., Zhu C., Wu M., Zheng F., Gao Y., Niu H. (2021). J. Mater. Sci..

[cit41] Vignesh S., Ahmad K., Oh T. H. (2025). Biosensors.

[cit42] Li Z., Xu J., Ding X., Zhu H., Wu J. (2025). Nanomaterials.

[cit43] Zhao D., Timmons D. J., Yuan D., Zhou H.-C. (2011). Acc. Chem. Res..

[cit44] Lu W., Wei Z., Gu Z.-Y., Liu T.-F., Park J., Park J., Tian J., Zhang M., Zhang Q., Gentle III T., Bosch M., Zhou H.-C. (2014). Chem. Soc. Rev..

[cit45] Manyani N., Siwatch P., Rana S., Sharma K., Tripathi S. K. (2025). Mater. Chem. Phys..

[cit46] Manyani N., Siwatch P., Rana S., Sharma K., Tripathi S. K. (2023). Mater. Res. Bull..

[cit47] Patel A., Patel S. K., Singh R. S., Patel R. P. (2024). Discover Nano.

[cit48] Pal B., Yang S., Ramesh S., Thangadurai V., Jose R. (2019). Nanoscale Adv..

[cit49] Zhong C., Deng Y., Hu W., Qiao J., Zhang L., Zhang J. (2015). Chem. Soc. Rev..

[cit50] Li M., Wang C., Chen Z., Xu K., Lu J. (2020). Chem. Rev..

[cit51] Xia L., Yu L., Hu D., Chen G. Z. (2017). Mater. Chem. Front..

[cit52] Wang Y., Zhong W.-H. (2015). Chemelectrochem.

[cit53] MacFarlane D. R., Forsyth M., Howlett P. C., Kar M., Passerini S., Pringle J. M., Ohno H., Watanabe M., Yan F., Zheng W., Zhang S., Zhang J. (2016). Nat. Rev. Mater..

[cit54] Zhou T., Gui C., Sun L., Hu Y., Lyu H., Wang Z., Song Z., Yu G. (2023). Chem. Rev..

[cit55] Zhang H., Liu X., Li H., Hasa I., Passerini S. (2021). Angew. Chem., Int. Ed..

[cit56] Hou Y., Hong H., Zhao Y., Yang X., Li D., Zhi C. (2025). Adv. Energy Mater..

[cit57] Misnon I. I., Aziz R. A., Zain N. K. M., Vidhyadharan B., Krishnan S. G., Jose R. (2014). Mater. Res. Bull..

[cit58] Wang J., Su Y., Lv S.-W., Sun L.-H. (2022). New J. Chem..

[cit59] Israr F., Chun D., Kim Y., Kim D. K. (2016). Ultrason. Sonochem..

[cit60] Maruthapandian V., Kumaraguru S., Mohan S., Saraswathy V., Muralidharan S. (2018). Chemelectrochem.

[cit61] Jin L., Liu Q., Sun W. (2013). Chin. Chem. Lett..

[cit62] Lestari W. W., Winarni I. D., Rahmawati F. (2017). IOP Conf. Ser.:Mater. Sci. Eng..

[cit63] Karuppiah K., Raju A., Natarajan A., Mohan S., subramani D., Rajendran K., Mahalingam V., Arumugam G., Prabhu S., Chiang K., Rajaraman V. (2025). Results Chem..

[cit64] Karuppiah K., Raju A., Natarajan A., Mohan S., Subramani D., Rajendran K., Mahalingam V., Arumugam G., Prabu S., Chiang K.-Y., Rajaraman V. (2025). Results Chem..

[cit65] Du P., Dong Y., Liu C., Wei W., Liu D., Liu P. (2018). J. Colloid Interface Sci..

[cit66] Gautam N. K., Shaalan N. M., Dalela S., Alvi P. A., Ahmed F., Brajpuriya R. K., Kumari K., Koo B. H., Sharma A., Kumar S. (2025). J. Phys. Chem. Solids.

[cit67] Mohamedien H. A., Mohamed F., Kamal S. M., Enaiet Allah A. (2026). RSC Adv..

[cit68] Nasit M., Kumari K., Yadav N., Koo B.-H., Dalela S., Vij A., Sharma A., Alvi P. A., Kumar S. (2026). J. Phys. Chem. Solids.

[cit69] Murugesan R. A., Nagamuthu Raja K. C. (2023). Mater. Res. Bull..

[cit70] Wang X., Mehandzhiyski A. Y., Arstad B., Van Aken K. L., Mathis T. S., Gallegos A., Tian Z., Ren D., Sheridan E., Grimes B. A., Jiang D., Wu J., Gogotsi Y., Chen D. (2017). J. Am. Chem. Soc..

[cit71] Lakshmi K. C. S., Vedhanarayanan B. (2023). Batteries.

[cit72] Gao S., Sui Y., Wei F., Qi J., Meng Q., He Y. (2018). J. Mater. Sci..

[cit73] Nasit M., Vij A., Kumari K., Koo B.-H., Dalela S., Alvi P. A., Brajpuriya R. K., Kumar S. (2025). J. Alloys Compd..

[cit74] Hashem Z. H., Abdel-Rahman L. H., Gómez-Ruiz S., Abdelhamid H. N. (2026). Catalysts.

[cit75] Lokhande A. C., Teotia S., Shelke A. R., Hussain T., Qattan I. A., Lokhande V. C., Patole S., Kim J. H., Lokhande C. D. (2020). Chem. Eng. J..

[cit76] Cheng C., Xu J., Gao W., Jiang S., Guo R. (2019). Electrochim. Acta.

